# Deep Learning Radiomics to Predict PTEN Mutation Status From Magnetic Resonance Imaging in Patients With Glioma

**DOI:** 10.3389/fonc.2021.734433

**Published:** 2021-10-04

**Authors:** Hongyu Chen, Fuhua Lin, Jinming Zhang, Xiaofei Lv, Jian Zhou, Zhi-Cheng Li, Yinsheng Chen

**Affiliations:** ^1^ Department of Neurosurgery/Neuro-Oncology, State Key Laboratory of Oncology in South China, Collaborative Innovation Center for Cancer Medicine, Sun Yat-sen University Cancer Center, Guangzhou, China; ^2^ Zhongshan School of Medicine, Sun Yat-sen University, Guangzhou, China; ^3^ Department of Medical Imaging, State Key Laboratory of Oncology in South China, Collaborative Innovation Center for Cancer Medicine, Sun Yat-sen University Cancer Center, Guangzhou, China; ^4^ Institute of Biomedical and Health Engineering, Shenzhen Institute of Advanced Technology, Chinese Academy of Sciences, Shenzhen, China

**Keywords:** glioma, deep learning, radiomics, magnetic resonance imaging, PTEN

## Abstract

**Objectives:**

Phosphatase and tensin homolog (*PTEN*) mutation is an indicator of poor prognosis of low-grade and high-grade glioma. This study built a reliable model from multi-parametric magnetic resonance imaging (MRI) for predicting the *PTEN* mutation status in patients with glioma.

**Methods:**

In this study, a total of 244 patients with glioma were retrospectively collected from our center (*n* = 77) and The Cancer Imaging Archive (*n* = 167). All patients were randomly divided into a training set (*n* = 170) and a validation set (*n* = 74). Three models were built from preoperative MRI for predicting PTEN status, including a radiomics model, a convolutional neural network (CNN) model, and an integrated model based on both radiomics and CNN features. The performance of each model was evaluated by accuracy and the area under the receiver operating characteristic curve (AUC).

**Results:**

The CNN model achieved an AUC of 0.84 and an accuracy of 0.81, which performed better than did the radiomics model, with an AUC of 0.83 and an accuracy of 0.66. Combining radiomics with CNN will further benefit the predictive performance (accuracy = 0.86, AUC = 0.91).

**Conclusions:**

The combination of both the CNN and radiomics features achieved significantly higher performance in predicting the mutation status of *PTEN* in patients with glioma than did the radiomics or the CNN model alone.

## 1 Introduction

Diffuse glioma is the most common primary brain tumor that mainly includes the World Health Organization (WHO) grades II, III (lower-grade glioma, LGG), and IV (glioblastoma, GBM). The WHO classification of central nervous system (CNS) tumors was updated in 2016 on the basis of the integrated diagnosis of molecular genetics (1). Phosphatase and tensin homolog (*PTEN*) is a common tumor suppressor gene that regulates the proliferation, survival, and other cellular processes by opposing the activation of phosphoinositide 3-kinase (PI3K)/protein kinase B (AKT/PKB) (2). The mutation status of *PTEN* is associated with poor prognosis (3, 4) and resistance to some treatments (5, 6) of multiple tumors, including glioma. Currently, the detection of PTEN status relies on genetic profiling approaches, requiring tumor tissue *via* surgical resection. Preoperative prediction of PTEN status has doubtful clinical benefits.

Previous studies have shown possible correlations between MRI and PTEN in GBM. GBM with *PTEN* mutations often occurs in the right frontal lobe (7). Cerebral blood volume and apparent diffusion coefficient (ADC) were also associated with PTEN status (8, 9). Although several studies have associated radiographic factors with the *PTEN* mutation status, the predictive precision is far from satisfactory. Recent advances in medical image analysis have allowed us to extract high-dimensional quantitative features from imaging. On the other hand, machine learning techniques permit predicting clinical outcomes using quantitative imaging features. Currently, there are two popular imaging-based machine learning approaches: radiomics and convolutional neural network (CNN). High-throughput radiomics features in MRI have shown their power in predicting *PTEN* mutations (10). Recent studies have also investigated the potential of radiomics features in predicting other molecular markers for glioma, such as isocitrate dehydrogenase (IDH) mutation (11), *O* ^6^-methylguanine-DNA-methyltransferase (MGMT) methylation status (12), and molecular subgroups (13, 14). However, radiomics depends on a handcrafted feature extraction pipeline. The handcrafted nature of radiomics features may be limited by our current understanding of medical images, which limits the potential of radiomics-based prediction methods.

Recently, many studies have shown the power of CNN in medical imaging (15, 16). CNN improved the handcrafted radiomics pipeline by automatically learning discriminative features directly from medical images. Recent studies have shown that deep CNNs can achieve better performance in tumor detection and diagnosis compared with other machine learning approaches and even human experts (17–19). CNN built from preoperative MRI or pathological images have been shown to be predictive of the IDH mutation status in glioma (20, 21). To our knowledge, little work has been done on associating CNN with the *PTEN* mutation status in glioma. Moreover, the region of interest (ROI) in most previous studies was manually delineated by specialists, which is costly and time-consuming. In recent years, deep learning-based models have become more reliable and accurate in the automatic segmentation of glioma from MRI (22–25). However, the performance of the automatic segmentation method has not been investigated and assessed in MRI-based prediction the *PTEN* mutation status in patients with glioma.

In this retrospective study, we investigated the benefits of combining both deep CNN and radiomics features extracted from MRI. The aim was to build a deep learning-based radiomics model for pretreatment prediction of the *PTEN* mutation status in glioma without any manual segmentation.

## 2 Materials and Methods

### 2.1 Patient Enrollment

**Figure 1:**
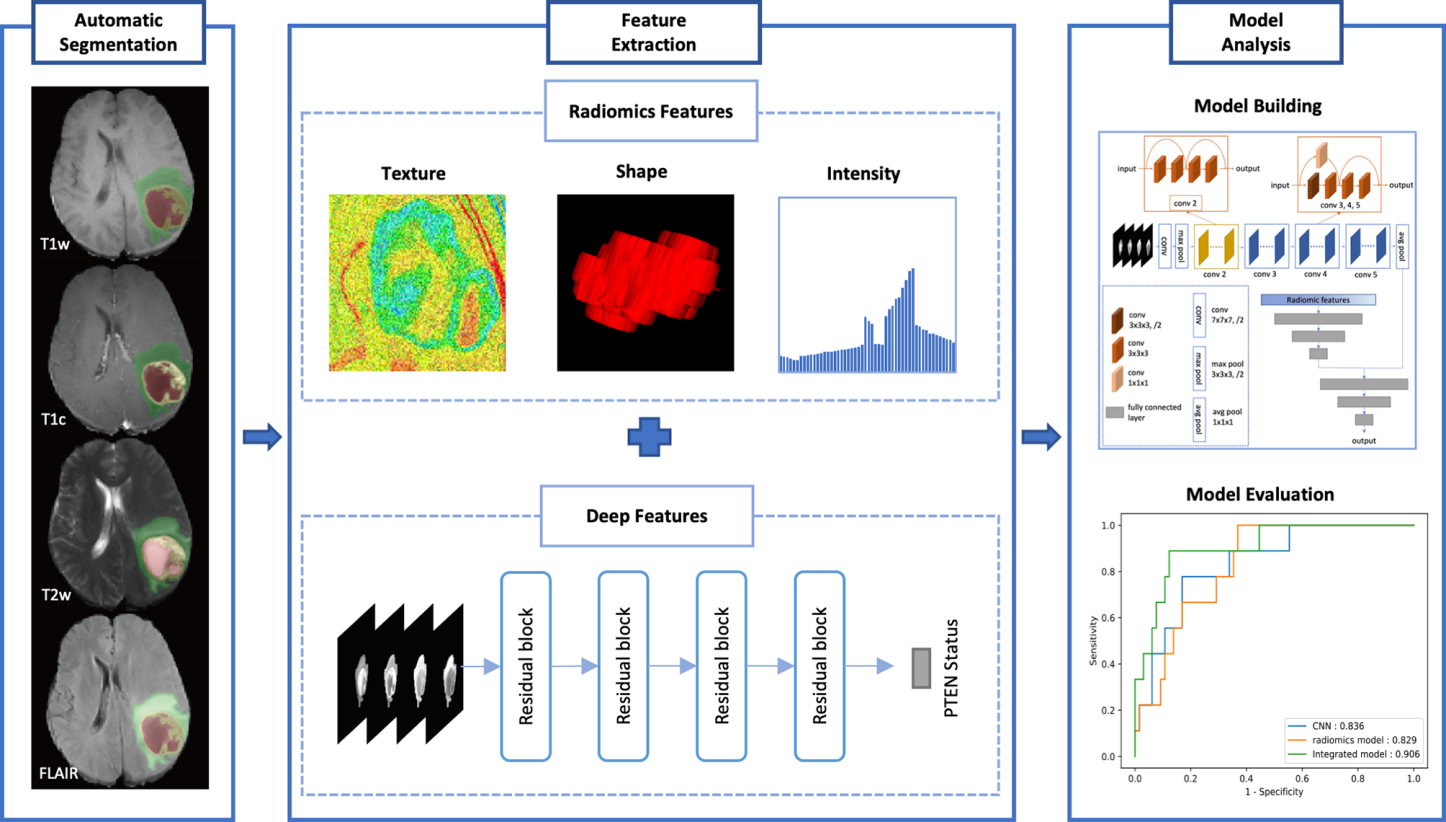
The design of our study.

In this retrospective study, 244 patients with glioma were recruited from The Cancer Imaging Archive (TCIA) and our center (Sun Yat-Sen University Cancer Center) between 2011 and 2016. TCIA is a publicly available database that removes, identifies, and hosts a large archive of medical images of cancer (www.cancerimagingarchive.net). Institutional Review Board approval for TCIA data was not required. Institutional Review Board approval from our center was obtained and informed patient consent was waived. All patients were randomly divided into two datasets. The training set of 170 patients comprised 114 from TCIA and 56 from our center. Another dataset of 74 patients comprising 53 from TCIA and 21 from our center was used for validation. The inclusion criteria were as follows: 1) patients with newly diagnosed histologically confirmed WHO grade I–IV glioma; 2) pretreatment MRI including T1-weighted, gadolinium contrast-enhanced T1-weighted, T2-weighted, and T2-weighted fluid-attenuated inversion recovery (T1w, T1c, T2w, and FLAIR, respectively); and 3) available *PTEN* mutation status. The *PTEN* mutation data of the TCIA patients were obtained from The Cancer Genome Atlas (TCGA), which includes genomics data corresponding to TCIA patients. The characteristics of the patients in the training and validation datasets are summarized in **Table 1**. The study design is shown in **Figure 1**.

**Table 1:** Patient and tumor characteristics of the study population.

Characteristic	TCIA	Local	*p*-value
No. of patients	167 (68.4%)	77 (31.6%)	
Age (years), mean (range)	51.7 (20–85)	40.8 (7–78)	<0.001
Sex			0.204
Female	84 (50.3%)	32 (41.6%)	
Male	83 (49.7%)	45 (58.4%)	
*PTEN*			0.072
Mutated	20 (12.0%)	16 (20.8%)	
Wild type	147 (88.0%)	61 (79.2%)	
WHO grade			<0.001
I	0 (0%)	10 (13.0%)	
II	64 (38.3%)	12 (15.6%)	
III	38 (22.8%)	10 (13.0%)	
IV	65 (38.9%)	45 (58.4%)	

TCIA, The Cancer Imaging Archive.

### 2.2 MR Imaging

All local MR images were acquired with 3.0-T MR imaging systems [uMR 780 (United Imaging), or Achieva (Philips), or Espree (Siemens Healthcare), or Discovery MR 750 (GE)]. T1w images were acquired at repetition time of 160–2,836.25 ms, echo time of 8–43 ms, and section thickness of 4.0–6.0 mm. T1c images were acquired at repetition time of 110–1,900 ms, echo time of 1.8–22 ms, and section thickness of 0.85–6.0 mm. T2w images were obtained with repetition time of 1,991.31–12,528.89 ms, echo time of 76.2–139.55 ms, and section thickness of 4.0–8.0 mm. FLAIR images were obtained with a repetition time of 2,000–9,600 ms, echo time of 20–141.3 ms, and section thickness of 4.0–6.0 mm.

### 2.3 *PTEN* Mutation Status Test

The *PTEN* mutation status of TCGA patients and the patients from our center was detected using whole-exome sequencing (WES). The tumor specimen that represents the characteristic was selected by experienced neurosurgeons for detection. Genomic DNA was extracted from fresh frozen tumor specimens and blood samples with a DNeasy Blood and Tissue Kit (Qiagen, Hilden, Germany). WES libraries were prepared using Agilent’s SureSelect Human All Exon V5 Kit (Agilent Technologies, Santa Clara, CA, USA) and sequenced on the Illumina HiSeq2000 Genome Analyzer platform (Illumina, San Diego, CA, USA). Sequencing reads were aligned to a human reference genome (UCSC hg19) using the Burrows–Wheeler Aligner (BWA) (26). Subsequent processing was performed using PICARD (http://picard.sourceforge.net), the Genome Analysis Toolkit (GATK), and VarScan 2 (27).

### 2.4 Image Pre-Processing and Tumor Subregion Segmentation

A pre-processing pipeline was applied on T1w, T1c, T2w, and FLAIR images for segmentation and image standardization. Firstly, skull stripping, N4ITK-based bias field correction, histogram matching-based intensity normalization, isotropic voxel resampling, rigid registration, and resizing to 240 × 240 × 155 pixels were performed using the BraTS Toolkit (23, 28, 29). The model from Zhao et al. (22) was implemented and the tumors were segmented into two subregions: solid tumor core (TC, comprising a contrast-enhancing area, a non- enhancing area, and necrotic portions, if any) and the whole tumor (WT, combining the tumor core and edema).

### 2.5 Radiomics Feature Extraction

**Figure 2:**
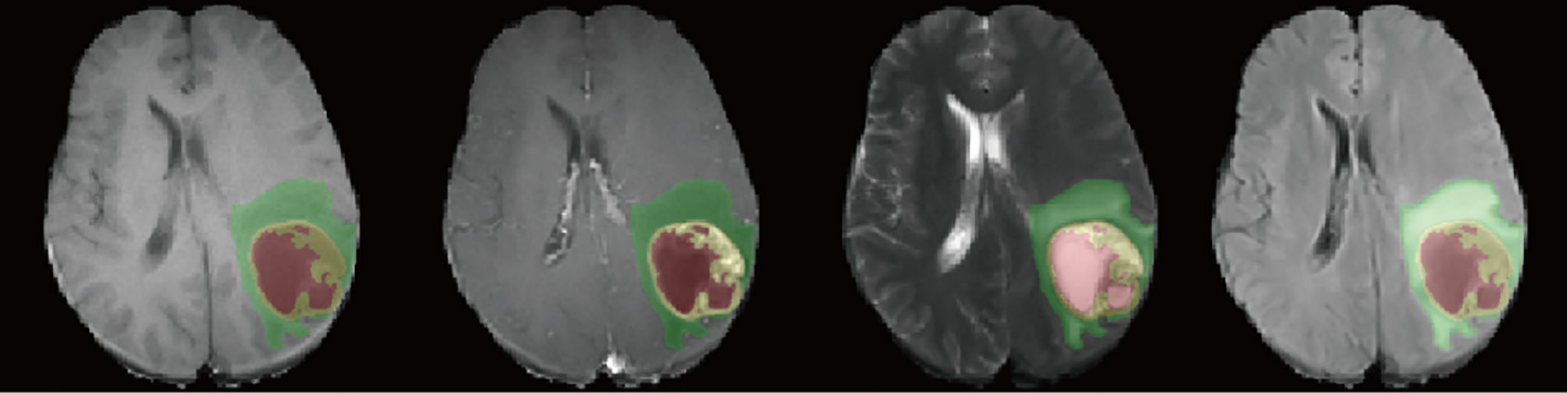
Example of the segmentation results for patients from our center. The four image modalities were T1, gadolinium contrast-enhanced T1 (T1c), T2, and fluid-attenuated inversion recovery (FLAIR), from *left* to *right*. *Yellow* represents the contrast-enhancing area. *Red* and *yellow* represent the tumor core (TC). The whole tumor (WT) contains all three labels.

Based on the segmented subregions, we extracted three groups of features according to recommendations of the Imaging Biomarker Standardization Initiative (IBSI) (30): 1) geometry features, 2) intensity features, and 3) texture features. The features were extracted within two extraction subregions from both the original image and a wavelet transformed image for each of the four MRI sequences. The wavelet filter decomposed the original image into eight decompositions. An example of the segmentation result is shown in **Figure 2**. For each subregion, 14 geometry features were extracted to describe the three-dimensional (3D) characteristics of the tumor shape. From the four MR modalities, and eight wavelet decompositions, 576 intensity features were extracted. These intensity features described the first-order distribution of the multi-regional intensities. The texture features were extracted using four methods, namely, the gray-level co-occurrence matrix (GLCM), gray-level run-length matrix (GLRLM), gray-level size zone matrix (GLSZM), and the neighborhood gray tone difference matrix (NGTDM). Two thousand four hundred texture features were computed from four MRI sequences, and eight decompositions, describing the patterns or the high-order distributions of the intensities. Finally, for each patient and subregion 2,900 quantitative features were extracted. All the calculations were conducted using a python package: PyRadiomics, version 3.0.1 (31).

### 2.6 VASARI Feature Extraction

Visually Accessible Rembrandt Images (VASARI) features were a controlled vocabulary of well-defined radiographic features (https://wiki.nci.nih.gov/display/CIP/VASARI), which aims to describe the morphology of glioblastoma on MR images. For comparison, we also extracted 26 VASARI features for the prediction of the *PTEN* mutation status. These features were measured by one neuroradiologist (H-YC) with 1 year’s experience in neuroimaging and neurosurgical oncology and confirmed by a neurosurgeon (F-HL) with 10 years’ experience in neurosurgical oncology. The reviews and measurements were conducted using an open-source software, ITK-SNAP, version 3.8.0 (32).

### 2.7 Prediction Model Construction

#### 2.7.1 ResNet Model

CNN can automatically learn discriminative features from images using multiple convolutional layers. The residual deep neural network (ResNet) is a popular CNN architecture that is widely used in object detection and image classification tasks. Here, a 3D ResNet consisting of 18 layers was chosen as the network backbone. The WT images from four MRI sequences were resized, trimmed, and padded with zero into a shape of (4, 32, 224, 224) (modality, depth, height, width). To handle the data imbalance problem, each image from the *PTEN* mutated patients in the training set was randomly rotated within −15° to 15° twice. The reshaped images were used as the ResNet input. The ResNet output was a class probability vector as the prediction result for each patient. The network was trained with binary cross-entropy loss function and root mean square prop optimizer with a regularization weight of 0.001 and a batch size of 16. The learning rate was 0.001. All the parameters were initialized with Glorot initialization (33). The details of the ResNet are summarized in **Supplementary Table S1**. Similarly, another popular CNN architecture named VGGNet was also implemented. Here, an 11-layer 3D VGGNet with batch normalization was trained. For a fair comparison, all the training hyperparameters were the same as those of the ResNet model.

2.7.2 Radiomics Model

For comparison, we also built a prediction model using only the radiomics features. Firstly, using high-dimensional radiomics features, feature selection was performed. The maximal information coefficient of each feature was then calculated and the top 30% was selected. The selected features were used to build a four-layer fully connected neural network, where a sigmoid end was used to generate the output probability. The rectified linear unit (ReLU) was used as the activation function of all hidden layers. The dropout technique, which is an effective technique for the regularization and prevention of the co-adaptation of neurons, was used in the first two layers after linear transformation. The dropout probabilities were 20% and 50%. The network was trained with binary cross-entropy loss function and root mean square prop optimizer with a learning rate of 0.001, a regularization weight of 0.05, and a batch size of 32. Details of the radiomics model are shown in **Supplementary Table S2**. All the calculations were conducted with the python package scikit-learn, version 0.23.2 (34).

#### 2.7.3 Integrated Model Based on Both ResNet and Radiomics Features

**Figure 3:**
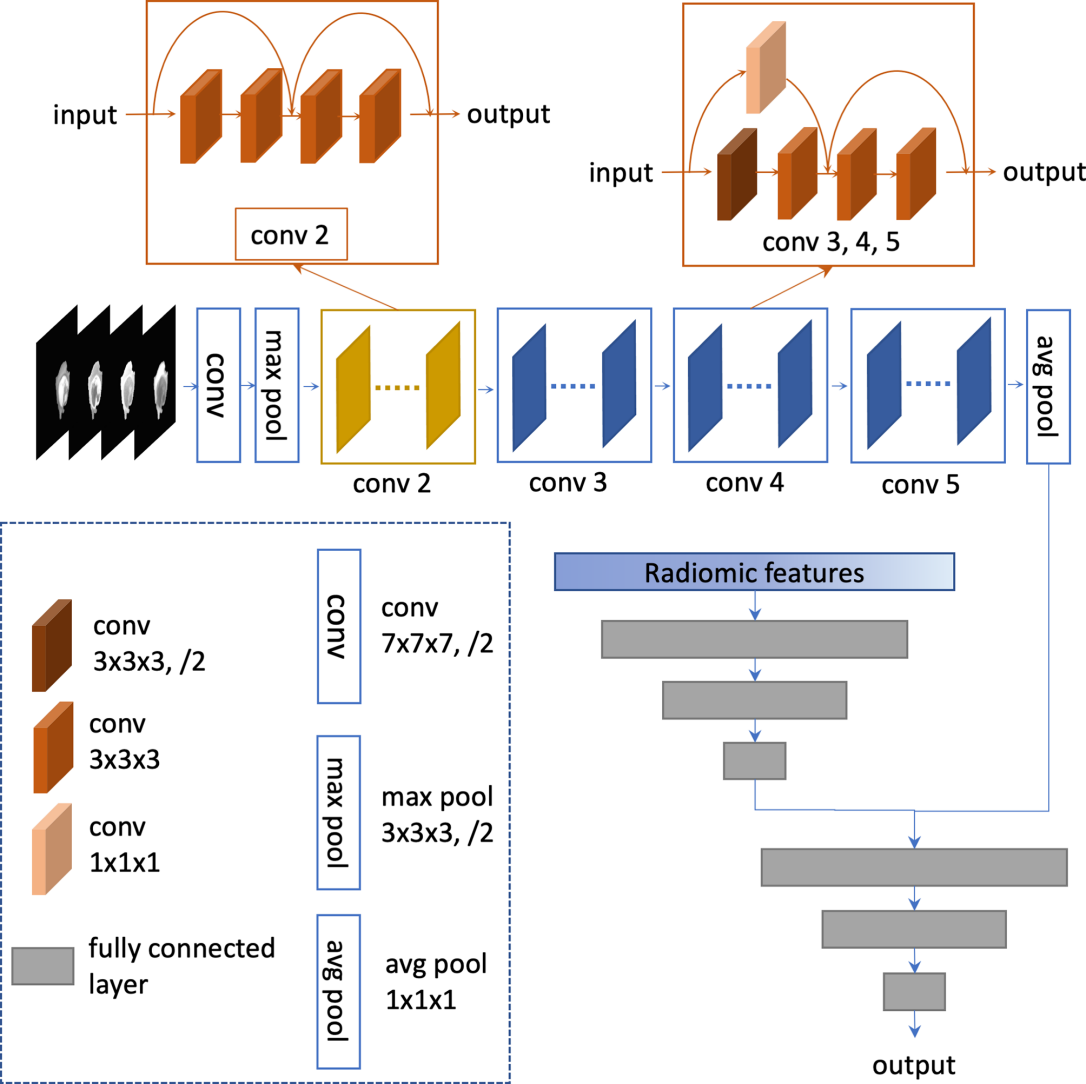
Architecture of the integrated model.

An integrated prediction model was built by combining the ResNet features and the radiomics features. The integrated model employed a four-layer fully connected network for mutation prediction. The ReLU was used as the activation function of hidden layers. The sigmoid end was used to yield the final prediction. The concatenation of the ResNet features extracted from the average pooling layer and the features extracted from the third layer of the radiomics model was fed into the integrated network. The network was trained with binary cross-entropy loss function and root mean square prop optimizer with a learning rate of 0.1, a regularization weight of 0.005, and a batch size of 16. The overall architecture of the final network is shown in **Figure 3** and **Supplementary Table S3**. The networks were implemented on PyTorch, version 1.7.0+cu110 (https://pytorch.org).

#### 2.7.4 VASARI Model

For further comparison, a VASARI model was built. For a fair comparison, the 26 VASARI features were fed into a four-layer fully connected neural network, the same as the radiomics model.

### 2.8 Statistical Analysis

All prediction models were trained on the training set and evaluated on the validation set. The predictive performance was assessed by accuracy (ACC), the area under the receiver operating characteristic curve (AUC), positive predictive value (PPV), and negative predictive value (NPV). The AUCs between models were statistically compared using the DeLong test (35). Furthermore, bootstrap resampling was performed to assess the average performance of all prediction models in terms of the AUC. Here, 100 bootstrapping repetitions were used with the training set of 170 patients and the validation subset of 74 patients. In each iteration, the model training and validation processes were repeated on the resampled training and validation sets, respectively. All statistical analyses were performed with R software, version 3.6.3 (https://www.r-project.org/).

## 3 Results

**Figure 4:**
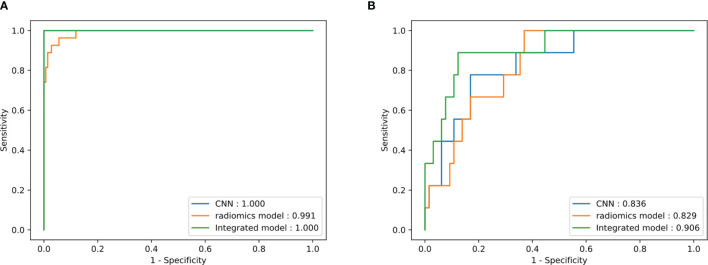
Receiver operating characteristic (ROC) curve of the three models in the training **(A)** and test **(B)** sets.

The characteristics of the patients are summarized in **Table 1**. The performances of the ResNet, radiomics, and integrated models in predicting the *PTEN* mutation status in the training and validation sets are summarized in **Table 2**. The receiver operating characteristic (ROC) curves in the training and validation sets are shown in **Figure 4**. Among all models, the integrated model showed the best performance, with the highest ACC of 86.5%, the highest AUC of 0.906, and the highest PPV of 87.7% in the validation set. The AUC of the integrated model was significantly higher than that of both the ResNet and radiomics models (DeLong *p* = 0.024 and 0.048, respectively, one-tailed). The ResNet model achieved an ACC of 81.1% and an AUC of 0.836, which were higher than those of the radiomics model, which had an ACC of 66.2% and an AUC of 0.829. The difference between the AUCs of the ResNet model and the radiomics model was not significant (DeLong *p* = 0.46, one-tailed).

**Table 2:** Summary of the performance of the CNN, radiomics, and integrated models in predicting the mutation status of PTEN in the training and validation datasets.

Model	Index	Training	Validation
ResNet	AUC	1.000 (1.000–1.000)	0.836 (0.707–0.965)
	ACC (%)	99.4	81.1
	PPV (%)	100 (97.5–100)	83.1 (71.7–91.2)
	NPV (%)	96.3 (81.0–99.9)	66.7 (29.9–92.5)
Radiomics model	AUC	0.991 (0.980–1.000)	0.829 (0.718–0.940)
	ACC (%)	94.1	66.2
	PPV (%)	94.4 (89.3–97.6)	63.1 (50.2–74.7)
	NPV (%)	92.6 (75.7–99.1)	88.9 (51.8–99.7)
Integrated model	AUC	1.000 (1.000–1.000)	0.906 (0.807–1.000)
	ACC (%)	99.4	86.5
	PPV (%)	100 (97.5–100)	87.7 (77.2–94.5)
	NPV (%)	96.3 (81.0–99.9)	77.8 (40.0–97.2)

Statistical quantifications were demonstrated with 95% confidential interval (CI), when applicable.

CNN, convolutional neural network; ACC, accuracy; AUC, area under the receiver operating characteristic curve; PPV, positive predictive value; and NPV, negative predictive value.

For comparison, the VGGNet model achieved an AUC of 0.591 in the validation set, which was numerically lower than that of the ResNet model. A significant difference between the AUCs of the VGGNet and ResNet models was found (DeLong *p* = 0.033). The VASARI model achieved an AUC of 0.755 in the validation set, which was much lower than that of either the CNN or the radiomics model.

The bootstrap-corrected AUCs in the validation set were 0.801 for the ResNet model, 0.824 for the radiomics model, 0.893 for the integrated model, 0.573 for the VGGNet model, and 0.728 for the VASARI model. The bootstrap-corrected results demonstrated the stability of our models for different data splitting.

## 4 Discussion

Medical images have the characteristic of having a huge amount of data with similar and standardized patterns. This characteristic indicates the potential of applying quantization and machine learning in medical images. Quantization of medical images can assist in clinical decision-making. With the rise of the concept of radiomics (36), high-throughput quantization of medical images is becoming possible. An effective radiomics analysis relies on the extraction and selection of prior known features. On the one hand, the extraction of high-throughput features might lead to problems of dimensionality and overfitting. On the other hand, radical feature selection might cause underfitting. CNN can automatically extract predictive features and transform them layer by layer. Recently, CNN-based models have achieved diagnostic accuracy and become clinically applicable in dermatology (17), ophthalmology (18), and gastroenterology (19), which have not been attained by radiomics approaches.

Although deep learning has outperformed radiomics, a huge number of data are needed for training and preventing overfitting. However, glioma is a relatively low-prevalence tumor, which accounts for only 2% of all primary tumors (37), and *PTEN* mutated patients are less than one-fourth of glioma patients (38). Even data enhancement may balance the data distribution, to some extent; the limited size of data restricts a variety of deep learning features, while prior known radiomics features can enhance the performance of a CNN-based model.

Glioma is the most common primary brain tumor. The prognosis and treatment of glioma are highly correlated with biomarkers (1). Previous studies have shown the promising ability of machine learning in predicting biomarkers and the survival of glioma patients using MRI. Lu et al. (39) showed the ability to predict the IDH mutation and 1p/19q co-deletion status, two classic biomarkers of glioma, with radiomics, achieving AUC values between 0.922 and 0.975. In the study by Han et al. (40), the effect of combining CNN features with radiomics using the Cox model was demonstrated. For predicting PTEN status, Ryoo et al. (8) proposed a radiographic feature, the normalized relative tumor blood volume (nTBV), where the AUC reached 0.674. Radiomics was also applied by Li et al. (10), obtaining an AUC value of 0.787. Although previous studies have shown the power of radiomics in predicting glioma molecular subtypes, its value in predicting PTEN status has only been seldom investigated. To the best of our knowledge, although MRI-based machine learning approaches have been demonstrated useful in predicting biomarkers of glioma, only a few studies have evaluated the value of CNNs in biomarker prediction, especially on the mutation status of *PTEN*. Meanwhile, no study has evaluated the combined effects of radiomics features and CNN with a deep learning-based model.

In this study, we built an integrated model from multi-parametric MRI and multi-regional radiomics features to predict the mutation status of *PTEN* in patients with glioma. The integrated model outperformed the CNN and radiomics models. Furthermore, unlike most previous studies, we did not merely include glioblastoma patients but also patients with other classifications of gliomas since the pathological diagnosis is unknown before surgery and our goal was to predict the *PTEN* mutation status before surgery. In this retrospective study, we firstly developed a CNN based on WT images and a fully connected neural network based on radiomics features for preoperative *PTEN* mutation status prediction. Additionally, we concatenated the CNN features from the full connection layer of the ResNet with the transformed radiomics features from the last but two layers of the radiomics model as supplements to the auto-extracted features.

In our study, although the CNN model showed higher ACC and AUC values than did the fully connected neural network based on radiomics features on both the training and validation sets, the difference in the AUC values was not significant (DeLong *p* = 0.050 and 0.462, respectively, one-tailed). By combining the radiomics features with the CNN features, the performance was further enhanced, and the improvement in the AUC was significant when compared with that of the CNN and radiomics models (DeLong *p* = 0.024 and 0.048, respectively, one-tailed).

Safe maximal resection is of utmost importance for glioma patients, while several reasons, such as a close relationship between the tumor and functional areas or vessels, may limit the extension of resection. It has been reported that neoadjuvant chemotherapy might be able to shrink glioma (41). Therefore, predicting biomarkers before surgery is necessary and clinically beneficial when the diagnosis, classification, treatment, and prognosis are all highly correlated with biomarkers. Especially, PTEN is a classic biomarker across multiple tumor types, including glioma (3). Mutations in *PTEN* will lead to a significantly shorter overall survival of glioma patients. The PTEN pathway may relate to radiation sensitivity and anti-angiogenic treatment resistance or serve as a therapeutic target (5, 6, 42, 43). Thus, researchers have briefly tried predicting mutations in *PTEN* noninvasively. During the pre-radiomics era, researchers mainly focused on some quantifiable factors and contrast agents (8, 44). With the rise of the concept of radiomics, high-throughput features have shown their ability to predict PTEN status (10). However, an AUC of 0.787 is far from satisfactory and limits further studies based on the preoperative PTEN status. By extending radiomics features with deep learning features, as our approach has described, a more precise prediction can be made. In our study, we recruited more patients and included all gliomas instead of only glioblastoma, making the model more robust and clinically translatable.

Our study has several limitations. Firstly, due to the population size, there was no independent test dataset. To further evaluate the robustness of the deep learning-based model, we will try to recruit an independent test dataset from lesser known centers. Moreover, the interpretability of deep learning-based networks is always a problem. Although we showed the efficiency of the CNN features, further descriptions of the mechanism of CNN features are highly required. In addition, with the advance of medical imaging, novel modalities such as dynamic susceptibility contrast-enhanced perfusion MRI are generally applied, which might provide extra factors for further improving the predictive precision.

## 5 Conclusion

In conclusion, the automatic CNN-based model allowed an accurate prediction of the mutation status of *PTEN* from preoperative MRI in patients with glioma, which achieved higher AUC, PPV, and NPV values compared to the radiomics model. Further combination of both the CNN and radiomics features achieved significantly higher AUC, PPV, and NPV values than did the radiomics or the CNN model alone.

## Data Availability Statement

The raw sequencing data have been uploaded to the Genome Sequence Archive (GSA) for human in the BIG Data Center, Beijing Institute of Genomics (BIG), Chinese Academy of Sciences, under the accession number HRA001024. The clinical data in this study has been deposited in the Research Data Deposit (RDD) under the RDD number RDDB2021959930.

## Ethics Statement

The studies involving human participants were reviewed and approved by the Ethics Committee of Sun Yat-sen University Cancer Center (approval number: GZR2021-340). The patients/participants provided written informed consent to participate in this study.

## Author Contributions

YC and Z-CL conceived and designed the study. J-MZ, XL, and JZ collected the molecular pathology and image data and performed pre-processing. HC and FL analyzed the data and performed the statistical analysis. HC wrote the manuscript. All authors contributed to the article and approved the submitted version.

## Funding

This work was supported by the National Natural Science Foundation of China (no. U20A20171), Youth Innovation Promotion Association of the Chinese Academy of Sciences (2018364), and the Guangdong Key Project (2018B030335001).

## Conflict of Interest

The authors declare that the research was conducted in the absence of any commercial or financial relationships that could be construed as a potential conflict of interest.

## Publisher’s Note

All claims expressed in this article are solely those of the authors and do not necessarily represent those of their affiliated organizations, or those of the publisher, the editors and the reviewers. Any product that may be evaluated in this article, or claim that may be made by its manufacturer, is not guaranteed or endorsed by the publisher.
