# Supplementary material for: Deep Learning Radiomics to Predict PTEN Mutation Status From Magnetic Resonance Imaging in Patients With Glioma
Source: Front Oncol. 2021 Oct 4;11:734433. doi: 10.3389/fonc.2021.734433 (PMC8521070; doi:10.3389/fonc.2021.734433)
Supplement: Supplementary file 1 [file Table_1.docx]

Table S1 The ResNet configuration used in our study. The network had four Basic Blocks that have 2 layers. Each layer consisted of two convolution layer. Before the first Basic Block, a  convolution with stride 1✕2✕2 followed by a  max pooling with stride 2 was performed on the input images. After the last Basic Block, a  adaptive average pooling was performed, and a sigmoid node was used to yield the final output. The output size of feature maps at each layer was shown

| Layers | Output size | Detail |
| --- | --- | --- |
| Convolution | 16 ✕ 112 ✕112 | 7 ✕ 7 ✕ 7, 64, 1 ✕ 2 ✕ 2 stride |
| Pooling-1 | 16 ✕ 112 ✕112 | 3 ✕ 3 ✕ 3 max pool, stride 2 |
| Conv-1 | 8 ✕ 56 ✕ 56 | 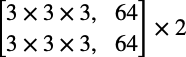 |
| Conv-2 | 4 ✕ 28 ✕ 28 | 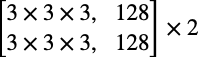 |
| Conv-3 | 2 ✕ 14 ✕ 14 | 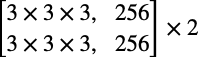 |
| Conv-4 | 1 ✕ 7 ✕ 7 | 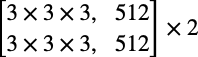 |
| Pooling-2 | 1 ✕ 1 ✕ 1 | 1 ✕ 1 ✕ 1 adaptive average pool |
| Fully connected | 2 |  |

| Layers | Output size |
| --- | --- |
| Linear-1 | 1024 |
| Linear-2 | 256 |
| Linear-3 | 64 |
| Linear-4 | 2 |

| Layers | Output size |
| --- | --- |
| Linear-1 | 256 |
| Linear-2 | 64 |
| Linear-3 | 16 |
| Linear-4 | 2 |

Table S2 The radiomics model configuration used in our study.

Table S3 The integrated model configuration used in our study.
